# A novel 3-methylthiophene additive to boost the performance and stability of perovskite solar cells

**DOI:** 10.1039/d1ra01236c

**Published:** 2021-03-10

**Authors:** Sadeer M. Majeed, Duha S. Ahmed, Mustafa K. A. Mohammed

**Affiliations:** Department of Applied Science, University of Technology Baghdad 100001 Iraq; Dijlah University College Al-Masafi Street, Al-Dora Baghdad 00964 Iraq mustafa_kareem97@yahoo.com +9647719047121

## Abstract

Perovskite solar cells (PSCs) have emerged as a practical candidate for new-generation photovoltaic devices to meet global energy demands. Recently, researchers' attempts have been focused on the crucial issues related to PSCs, *i.e.*, stability and performance. In this research, MAPbI_3_-based PSCs were prepared *via* a two-step deposition process. To boost the power conversion efficiency (PCE) of the prepared PSCs, an additive engineering approach was employed. A novel 3-methylthiophene (MTP) organic molecule was added to the methylammonium iodide (MAI)/isopropanol (IPA) solution precursor. The additive improved the crystallinity of the perovskite layer, which indicates a more desirable film with lower surface defects and larger particle size. Modified PSCs reduced carries recombination rate at the interfacial of perovskite/hole transport layer (HTL), and the charge transport process is facilitated due to a desirable delocalized π-electron system of the MTP additive. The PCE of PSCs in the presence of MTP additive improved from 12.32% to 16.93% for pristine devices. Importantly, MTP-based PSCs showed higher ambient air stability due to the hydrophobic structure of MTP compared to pristine PSCs.

## Abstract 2

Perovskite solar cells (PSCs) have emerged as a practical candidate for new-generation photovoltaic devices to meet global energy demands.

## Graphical abstract

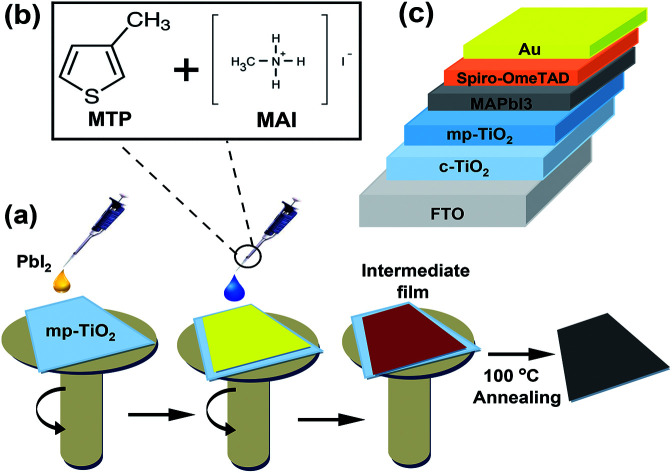

## 1. Introduction

In recent years, global warming concerns, and consequently global climate changes,^1^ have attracted the attention of many governments worldwide. Simultaneously, with an increase in the Earth's population, global energy demands (GEDs) have increased. These days, fossil fuels, which increase heat-trapping greenhouse gas levels in the Earth's atmosphere, are known as the primary source to meet GEDs. To solve this issue, researchers have suggested green and renewable energy sources, for instance, solar energy, wind energy, and hydro-energy. Typically, solar cell technology has provided a green energy source to energy demands.^2–5^ Recently, next-generation solar cell technologies have emerged to develop efficient and low-cost solar cells. Organic–inorganic PSCs are the most promising technology to facilitate efficient and low-cost solar cell development.^6–8^

Unique optoelectronic characteristics of MAPbI_3_ films, for instance, high absorption in a broad spectrum, low exciton energy, tunable bandgap, long-length carrier diffusion, and the low recombination rate, make them favorable for photovoltaic (PV) applications.^9–11^ To date, different strategies have been used to boost the PV parameters of PSCs. Electron transport layer (ETL) and HTL modification,^12,13^ interfacial engineering at the interface of perovskite layer and charge transport layers,^14,15^ grain boundary passivation of perovskite film,^16^ additive engineering,^17^ and composition engineering of perovskite structure^18^ are some of the efficient methods to enhance the performance of PSCs.

However, the efficiency of PSCs with fast progress rose to a competitive value with silicon solar cells, but they suffer from intrinsic short-term stability. When the perovskite layer is exposed to humidity, light irradiation, or heat, the perovskite film starts to degrade. A routine degradation process in well-known MAPbI_3_ (CH_3_NH_3_PbI_3_) is CH_3_NH_3_PbI_3_ → CH_3_NH_2_↑ + HI↑ + PbI_2_. Encapsulation of PSCs is suggested to protect the perovskite layer from humidity and oxygen. Lee *et al.*^19^ used a thin-film encapsulation (TFE) method with a periodical structure of organic (poly(1,3,5-trimethyl-1,3,5-trivinylcyclotrisiloxane)) and inorganic (Al_2_O_3_) layers. They found that the TFE method could improve the stability of PSCs specifically. The encapsulated device maintained 97% of its initial PCE. However, this method increases the cost of the fabrication process of PSCs. In addition, the encapsulation process has its challenges.^20–22^ Besides, improving the crystallinity properties of perovskite layers can also address the stability of PSCs.^10^ Saidaminov *et al.*^23^ expressed that the origin of the lattice strain is due to the ionic size mismatch between the A cation and lead halide in the perovskite structure. They proved that Cd incorporation could remove strain lattice in the perovskite structure without introducing traps and boost ambient air stability of corresponding PSCs. Boosting the hydrophobic behavior of the perovskite layer is an alternative technique to address the poor humidity stability of PSCs.^24^ Shu *et al.*^25^ introduced phenyltrimethylammonium bromide (PTABr) to modify and boost the surface hydrophobicity of the perovskite. After the addition of PTABr to the perovskite film, unencapsulated PSCs retained 86% of their original PCE after keeping for two months in air conditions.

In this study, MAPbI_3_ PSCs were fabricated by a two-step deposition process. To boost the PV properties of the fabricated PSCs, a novel 3-methylthiophene (MTP) molecule was inserted into the MAI/IPA precursor solution. The suggested approach showed that the MTP additive enlarges the crystal grain size of the MAPbI_3_ layer and consequently suppresses the grain boundaries in the perovskite layer. It was also found that the PV parameters of MTP-treated devices show more improvement compared to devices without additives. The modified PSCs with the MTP additive showed more stable behavior in humid conditions, indicating the improved hydrophobic behavior of the MTP -based perovskite layer.

## 2. Experimental details

### 2.1. Materials

Chlorobenzene (CB), MAI, and lead iodide (PbI_2_) were provided from Merck. Fluorine tin oxide (FTO, 15 Ω sq^−1^) was provided from Solaronix. 4-Tertbutylpyridine (TBP), phenyl-C61-butyric acid methyl ester (PCBM), ethanol (40%), and bis(trifluoromethane)sulfonimide lithium salt (Li-TFSI, 99.95%) were obtained from Alfa Aesar. Note that all additional chemicals were obtained from Sigma-Aldrich.

### 2.2. Solution preparation

Compact TiO_2_ (c-TiO_2_) was prepared by adding 2.5 ml of ethanol and 35 μl HCl (2 M) to 350 μl of titanium(iv) tetra isopropoxide (in 2.5 ml ethanol) under stirring at 0 °C for 15 min. To prepare mesoporous TiO_2_ (mp-TiO_2_), TiO_2_ paste was dissolved in ethanol at a 1 : 5 ratio and stirred for one day in ambient air. PbI_2_ solution was prepared by dissolving 600 mg of PbI_2_ powder in 950 μl of *N*,*N*-dimethylformamide and 50 μl of dimethyl sulfoxide and stirred at 65 °C overnight. The MAI solution was prepared by dispersing 40 mg ml^−1^ of MAI powder in IPA and stirring for 20 min at room temperature (RT). For MAI/MTP mixture, an MTP additive was added to the MAI/IPA solution at different volume ratios (2.5%, 5%, and 7.5%) and mixed by stirring for 5 min at RT. The HTL solution was obtained by mixing 17.5 μl Li-TFSI in acetonitrile (520 mg ml^−1^) and 28.8 μl TBP to a 60 mM Spiro-OMeTAD in CB solvent.

### 2.3. Device fabrication

The cleaning process of FTO substrates was done as per a general routine in the literature.^26^ The c-TiO_2_ was deposited on FTO at 2000 rpm for 30 s, followed by annealing at 500 °C for 25 min. Then, mp-TiO_2_ layers were deposited at 4000 rpm for 60 s and baked at 450 °C for 40 min. For perovskite films, 1.2 M PbI_2_ was spin-coated at 3500 rpm, followed by drying at room temperature for 1 min and annealing at 100 °C for 3 min. A 40 mg ml^−1^ MAI solution was then loaded on the PbI_2_ layer for 5 s, followed by spin coating at 3000 rpm for 30 s. After that, intermediate perovskite films were annealed at 100 °C for 10 min to complete the perovskite layer fabrication. The HTL solution was spin-coated at 4000 rpm for 30 s on top of the perovskite layer. Finally, a 100 nm Au electrode layer was evaporated on the HTL.

### 2.4. Characterization

Ultraviolet-visible spectroscopy (UV-vis, Ocean Optics) and steady-state photoluminescence (PL) spectroscopy (Agilent Cary Eclipse Fluorescence) were employed to study the optical features of perovskite films. The structural properties of perovskites were investigated by X-ray diffraction (Shimadzu). The morphology of perovskites was investigated using scanning electron microscopy (SEM, VEGA3). Current density–voltage (*J*–*V*) characteristics of PSCs were studied using a Keithley instrument (Model 2400, AM 1.5G one Sun) under 100 mW cm^−2^ illumination. The devices were measured with a 2 mm × 4 mm mask. A contact angle test of the perovskite layers was characterized, utilizing Phoenix 300. The incident photon to-current efficiency (IPCE) spectra were acquired by an IPCE system (Newport, USA).

## 3. Results and discussion

**Fig. 1:**
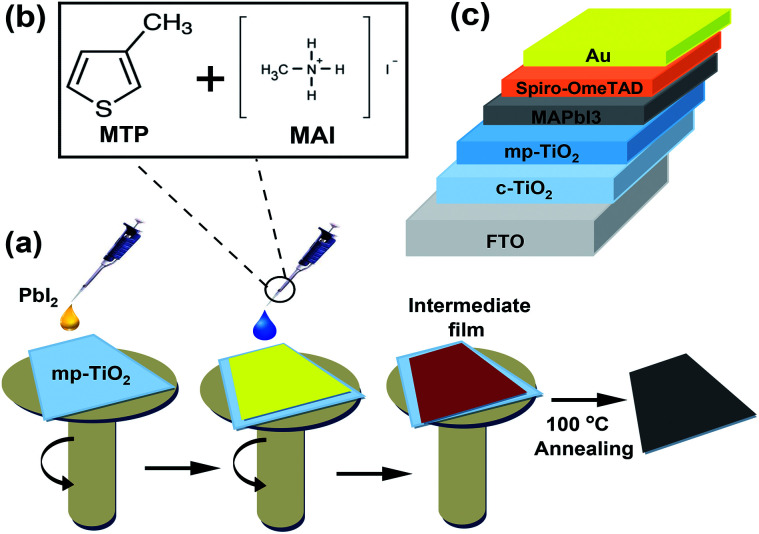
MTP additive assisted MAPbI_3_ fabrication process. (a) Schematic illustration of the procedure for fabricating MTP-modified MAPbI_3_ layer. (b) Chemical structures of MTP and MAI. (c) A scheme of the PSC device architecture (FTO/c-TiO_2_/mp-TiO_2_/MAPbI_3_/Spiro/Au).

For mesoporous PSCs, MAPbI_3_ perovskite films were fabricated *via* a two-step deposition process (see Fig. 1a). MTP additives in different contents (2.5%, 5%, and 7.5%) were used in the MAI precursor solution to assist the charge extraction process from perovskite and prevent the degradation of the corresponding devices. Fig. 1b demonstrates the chemical structures of MTP additive and MAI. The mesoporous PSC devices designed in this study were structured as FTO/c-TiO_2_/mp-TiO_2_/MAPbI_3_/Spiro-OMeTAD/Au (see Fig. 1c). A solution of dense yellow PbI_2_ was deposited onto mp-TiO_2_ ETL, followed by spin-coating MAI/MTP mixture. The as-prepared MAPbI_3_ layers were annealed to complete the crystallization process. Spin-coating of the Spiro-OMeTAD HTL and evaporation of the Au electrode completes the device architecture. The PCE of the subsequent PSCs was found to vary with the MTP amount.

**Fig. 2:**
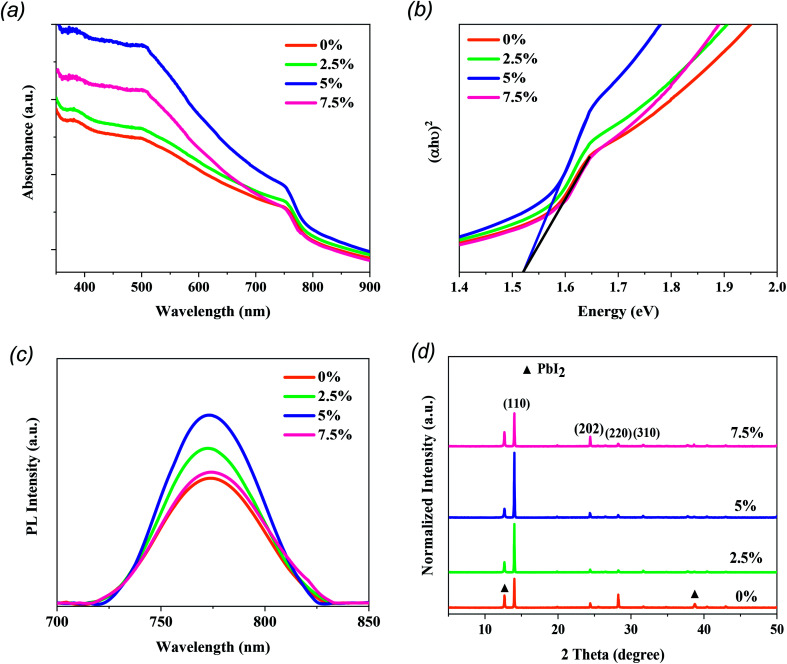
The characterization of MAPbI_3_ films modified with different amounts of MTP additive. (a) UV-vis absorption spectra. (b) Tauc plots of the corresponding films. (c) Steady-state PL spectra. (d) XRD patterns.

The impacts of the MTP additives on the optical and structural properties of the MAPbI_3_ photoactive layers were carefully studied by a range of measurements. As shown in Fig. 2a, the UV-vis absorption spectra reveal that the MAPbI_3_ layer with 5% MTP shows an optimum absorbance compared to the pure and 2.5% MTP-treated perovskite layers. Nevertheless, an excess amount of MTP (7.5%) leads to a lower absorbance due to lower crystallinity of the MAPbI_3_ layer (will be discussed later) and an inferior PSC performance. The enhanced UV-vis spectrum of MAPbI_3_ is in favor of a boosted *J*_sc_ when used in solar cells.^27^ The optical bandgap of pure and MTP treated perovskite films was measured by Tauc plot^28–30^ and is depicted in Fig. 2b. The bandgap (*E*_g_) of the MAPbI_3_ layers is the same as that of the ref. 3 and ^5^, according to UV-vis measurements. As seen in Tauc plots, the absorption spectra of MAPbI_3_ films show an identical bandgap of 1.52 eV, implying that the well-crystallized MAPbI_3_ films are deemed a direct *E*_g_ semiconductor. The steady-state PL spectra of pure and MAPbI_3_-containing various contents of MTP additives are presented in Fig. 2c. PL plots reveal identical PL peak position (773 nm) even when the concentration of MTP in MAI solutions increases, indicating that the MTP treatment and varying its content does not significantly alter the *E*_g_ of the fabricated MAPbI_3_ layers.^31^ Moreover, the PL intensity of the perovskite film with 5% MTP was improved, while that of 2.5% and 7.5% MTP merely enhanced. This indicates that the photoluminescence quenching effect is significantly improved. This means that the defect states of MAPbI_3_ with 5% MTP was suppressed, leading to the increased fill factor (FF) and efficiency of the PSC by reducing the recombination rate.^32^ The UV-vis spectra of MAPbI_3_ films demonstrate consistent characteristics of the PL measurements, where the perovskite with 5% MTP has the highest light-harvesting and charge extraction abilities compared with other MAPbI_3_ films.

The XRD patterns of MAPbI_3_ perovskites treated with MTP are illustrated in Fig. 2d. Note that all MAPbI_3_ layers are tetragonal crystals with reflection peaks at 14.03°, 24.41°, 28.25°, and 31.7° related to the (110), (202), (220), and (310) diffraction planes, respectively.^33^ By comparison, the perovskite film with 5% MTP additive reveals intensified peaks compared to the other films, indicating a highly crystalline MAPbI_3_ structure. Also, a high reflection peak of PbI_2_ located at 12.4° can be observed in the pure MAPbI_3_ layer, suggesting the presence of residual PbI_2_ and more decomposition of untreated MAPbI_3_ film during the fabrication process. With MTP treatment, the peak of PbI_2_ is significantly quenched, especially for the perovskite with 5% MTP additive. This implies that the more stabilized MAPbI_3_ can be achieved with MTP treatment.^34^ However, when the amount of MTP increases to 7.5%, MAPbI_3_ exhibits a higher signal of PbI_2_, and the crystallinity of MAPbI_3_ reduces. As stated in the UV-vis and PL results, excess amount of MTP has a diverse effect on the crystallinity and reduces the light absorption and charge extraction properties. No shift in the peaks nor new peaks are shown in the XRD results, indicating that the MTP treatment does not change the structure of the MAPbI_3_ layers. Combining the XRD results with the above findings from UV-vis and PL experiments, a conclusion can be drawn that the impact of MTP is essential. At optimized content (5%), an enhancement of crystallinity is observed, giving rise to the light-harvesting efficiency with low recombination rate in MTP treated film compared to the pristine film.

**Fig. 3:**
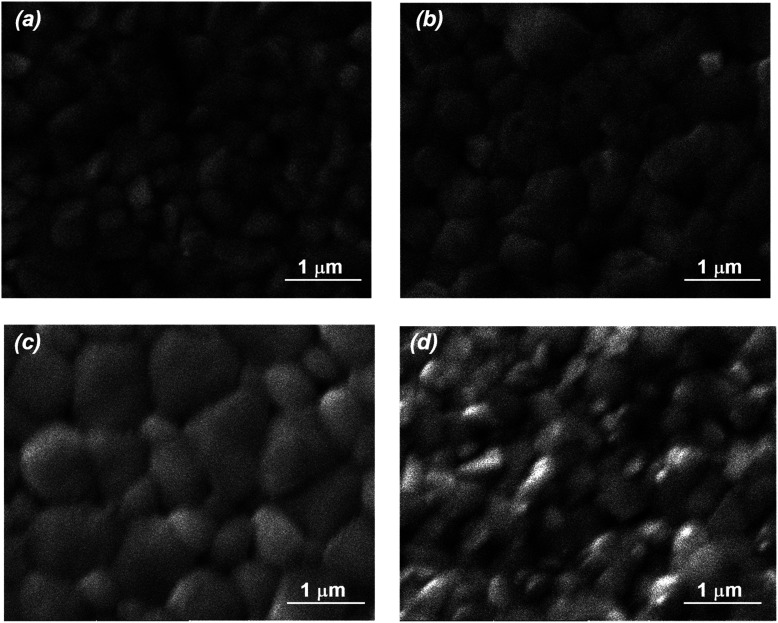
Surface characterization of MAPbI_3_ films using top-view FESEM. (a) Pristine film. (b) Treated with 2.5% MTP. (c) Treated with 5% MTP. (d) Treated with 7.5% MTP.

To further check the strengthening impact of MTP on perovskites, FESEM was employed to follow the MAPbI_3_ morphology change. Upon MTP modification, MAPbI_3_ layers show a considerably larger grain size than that of the pristine MAPbI_3_ layer (Fig. 3a–c), which is probably because the MTP additive may influence the crystal nucleation and crystallization kinetics of MAPbI_3_. Besides, the grain boundaries are suppressed from the non-uniform surface of the untreated perovskite layer with an increase in the MTP volume ratio, showing compact films with larger grain size and hence improved MAPbI_3_ crystallinity. It is well-known that humidity can permeate within the grain grooves to degrade the MAPbI_3_ layer.^35^ Therefore, increasing the grain size is an efficient strategy to stabilize the perovskite film. On the contrary, a perovskite film with smaller grains and some pinholes on its surface appeared in the 7.5% MTP treated film (Fig. 3d), indicating that a perovskite film with more grain boundaries, such as this film, is not desirable for photovoltaic application. In other words, excess MTP (7.5%) hinders the nucleation and development of a uniform MAPbI_3_ layer, resulting in smaller crystallite dimensions with many flaws on the surface. The variation of MAPbI_3_ layer morphologies in top-view FESEM images is consistent with XRD results with the variation of MTP amounts as well.

**Fig. 4:**
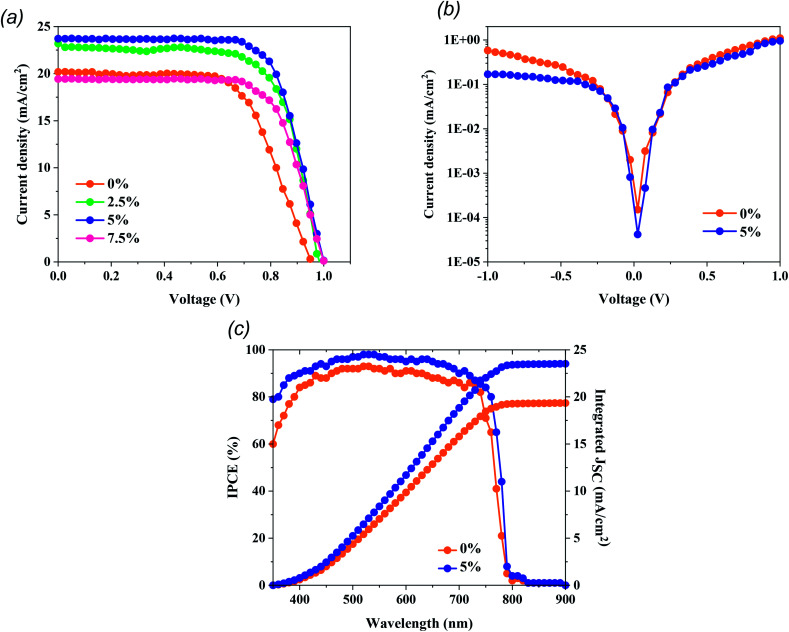
Performance of PSCs treated with different amounts of MTP. (a) *J*–*V* curves with reverse scan of PSCs. (b) Dark *J*–*V* curves of PSC based pristine and 5% treated MAPbI_3_ films. (c) IPCE spectra of PSC based pristine and 5% treated MAPbI_3_ films.

To probe the PV performance based on our treatment approach, we constructed n–i–p mesoporous PSCs employing MAPbI_3_ films as the photoactive materials. As presented in Fig. 4a and Table 1, the MAPbI_3_-based PSCs exhibited increased PV parameters after MTP treatment. *J*–*V* measurements revealed that cell performance improves with MTP amount until the volume ratio = 5%. The best PSC was fabricated with 5% MTP modification and yielded a PCE of 16.93%, a *J*_sc_ of 23.72 mA cm^−2^, a *V*_oc_ of 1.00 V, and an FF of 71.4%. We attribute this enhancement to the better crystallization process of perovskite, passivation of the grain boundaries, and lower charge recombination due to the MTP treatment, which is consistent with the trend shown in XRD, PL, and FESEM results. As described in the MAPbI_3_ characteristics, the perovskite film with appropriate MTP additive depicts improved light-harvesting, contributing to generating more photo-induced electrons and holes. The MTP treated perovskite showed fewer boundaries and defects. Therefore, the number of carrier recombination sites is significantly suppressed. For too much MTP additive (7.5%), the PV performance of PSCs suppresses to a lower PCE of 13.65%. As exhibited in Fig. 4b, the dark current density of the PSC with MTP-modifying MAPbI_3_ is lower than that of PSC with pristine MAPbI_3_, signifying that the leakage current of PSC with MTP treatment is decreased, resulting in the retardation of charge recombination and suppressed defects of perovskite.^36^ The corresponding IPCE spectra of champion PSCs with pristine and optimized MAPbI_3_ films were also recorded and are illustrated in Fig. 4c. As seen, the integrated current densities from the pristine and treated PSCs are 19.3 mA cm^−2^ and 23.4 mA cm^−2^, respectively, which corroborate with the *J*_sc_ values from the *J*–*V* sweep measurement. Furthermore, the device with the MTP additive shows higher IPCE than the pristine one, which agrees well with the highest *J*_sc_. These findings are again consistent with the features of MTP treatment, which contribute to better crystallized MAPbI_3_ and effective charge injection.

**Table 1:** PV parameters of PSCs with different amounts of 3-methylthiophene (MTP) additive in perovskite precursor

Device		*V*_oc_ (V)	*J*_sc_ (mA cm^−2^)	FF (%)	PCE (%)
0%	Average	0.939	19.22	62.36	11.25
	Best	0.962	20.19	63.50	12.32
2.5%	Average	0.964	21.95	67.40	14.37
	Best	0.987	23.20	68.20	15.61
5%	Average	0.990	23.03	70.02	16.00
	Best	1.000	23.72	71.40	16.93
7.5%	Average	0.985	19.19	69.13	13.06
	Best	1.000	19.44	70.2	13.65

**Fig. 5:**
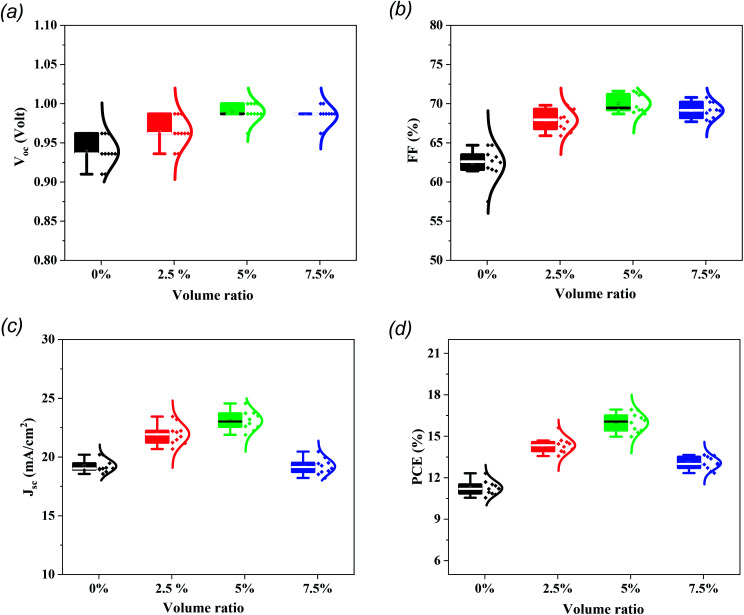
The statistical distribution of (a) *V*_oc_, (b) FF, (c) *J*_sc_, and (d) PCE of PSCs with various volume ratios of MTP additive in perovskite precursor. For each group, 10 devices were monitored.

With regard to PSC reproducibility, 10 cells were constructed and *J*–*V* measurements were carried out under AM 1.5G illumination conditions. The statistical distribution of PV parameters is presented in Fig. 5 and it is clear that the cells modified with 5% MTP reveal excellent reproducibility and a higher average PCE (16.0%) than those with pristine cells (11.25%). Additionally, the interval statistical distribution of 5% MTP-modified cells is narrower than that of the unmodified cells, which indicates good reliability of MTP function and agrees well with the findings from the champion PSC.

**Fig. 6:**
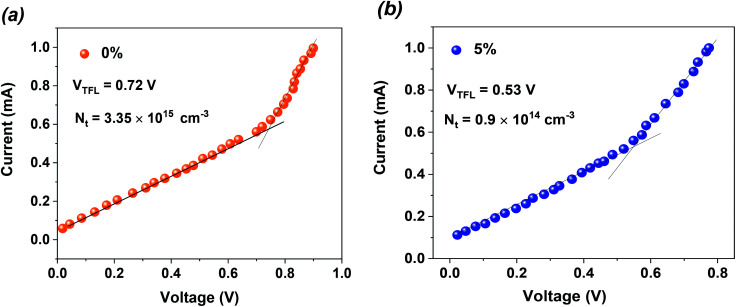
Dark current–voltage curves of the electron-only devices for (a) pure MAPbI_3_ and (b) MTP-treated MAPbI_3_ displaying *V*_TFL_ kink point behavior.

As shown above, additive engineering significantly increases the PCE of PSCs, and the PSCs based on 5% MTP achieve most outstanding performance. Thus, we performed additional measurements on devices based on pure MAPbI_3_ and 5% MTP treated MAPbI_3_ perovskite films. To evaluate the trap-state density (*N*_t_) of perovskites by space-charge-limited current (SCLC) measurement, we prepared the electron-only structures with the configuration of FTO/c-TiO_2_/mp-TiO_2_/MAPbI_3_/PCBM/Au, and measured the dark current–voltage curves, as depicted in Fig. 6a and b. According to the below formula, *N*_t_ can be calculated by the trap filled limit voltage (*V*_TFL_).
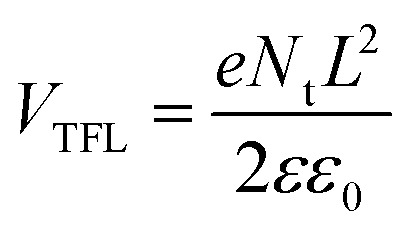
where *e* is the elementary charge of the electron, *L* is the thickness of the perovskite film (800 nm), *ε* is the relative dielectric constant (MAPbI_3_ is 32), and *ε*_0_ is the vacuum permittivity. The *V*_TFL_ values of pure and 5% MTP treated MAPbI_3_ films are 0.72 V and 0.53 V, with the corresponding *N*_t_ of 3.35 × 10^15^ cm^−3^ and 0.9 × 10^14^ cm^−3^, respectively. The significantly lower *N*_t_ indicates that the defects have indeed been reduced by the introduction of MTP, which may be attributed to the better quality of modified-MAPbI_3_ than pure film, resulting from smoother surfaces and the preferential crystal orientation in perovskite films. It is well known that the defects in perovskite film would hinder the mobility of charge carriers. Therefore, the reduced trap density may promote the carrier mobility in the perovskite film.^11^

**Fig. 7:**
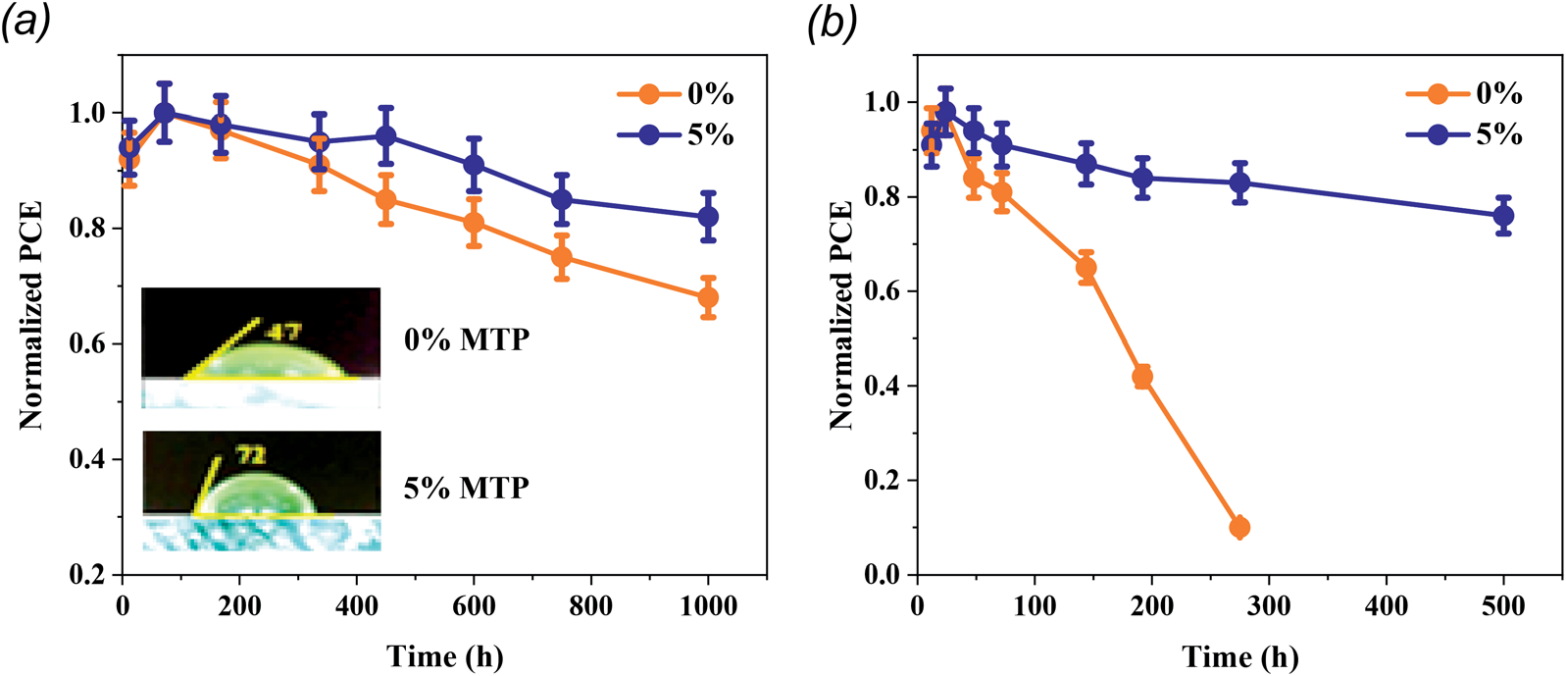
The stability test of unencapsulated PSCs with and without MTP additive at room temperature. (a) Stored in a dry box (<20% RH) (b) and those kept in ambient air with a RH of 30–50% in the dark conditions. Four devices of each group were utilized for the stability tests and 5% of the error bars were adopted. Inset of (a) represents the contact-angle measurement of perovskite films with and without additive.

The pinholes centered at the surface and internal grain boundaries in the MAPbI_3_ layer always impede the charge transfer and become recombination sites, reducing the carrier lifetime. In this context, these sites are inclined to become the adsorption centers for moisture and O_2_, therefore causing the suppression of PCE and the stability of PSCs.^37^ High-quality perovskite film is substantial to resist erosion by moisture and to stabilize the PSCs. In order to investigate the hydrophobicity of pristine and 5% MTP-treated MAPbI_3_ layers, water-contact angles were measured (inset of Fig. 7a). By comparing the water-contact angles on MAPbI_3_ layers, the contact angle of the pristine and MTP modified MAPbI_3_ is 47° and 72°, respectively, indicating that the hydrophobicity of MAPbI_3_ film greatly enhanced after MTP modification. In addition, previous reports have shown that there is a strong relationship between the wetting capability of water on MAPbI_3_ and PSC stability, particularly the contribution to the erosion resistance capability and the overall stability of PSC in moisture conditions.^12,38^

To elucidate the operational stability of our enhanced MAPbI_3_-based solar cells, we performed a series of measurements on unencapsulated PSCs at room temperature. As demonstrated in Fig. 7a, the stability of MTP-treated PSCs reveals a higher stabilized PCE. The MTP based PSCs maintain 83% of their initial PCE after storing in a dry box (<20% RH) for 1000 h, whereas pristine PSCs maintain only 64% of their original PCE. Most importantly, we investigated the effect of aging time on unencapsulated PSCs in ambient conditions with RH of 30–50% (Fig. 7b). The pristine PSCs exhibit a fast decline of PCE (degrades below 10% in less than 300 h). In contrast, the MTP-treated PSCs show a long lifetime, attaining over 80% of their original PCE after 500 h, which further verifies that the MTP treatment strategy brought about significant enhancements in the long-term stability of devices. The more stable behavior of the treated PSCs is due to the improved hydrophobic behavior of the perovskite films after the addition of MTP additive, as shown in the inset of Fig. 7a.

It is worth noting that the achieved findings revealed that the design of PSCs employing the MTP treatment in ambient air could enhance the PCE and stability of the MAPbI_3_-based solar cells, which is a step forward in PSC commercialization. Based on the comparison of the results listed in Table 2, it can be argued that the most beneficial approach for MTP usage in PSCs is incorporating it into the MAI solution, as reported here. As shown, the MTP additive was compared with ethylammonium chloride (EACl), 1,8-diiodooctane (DIO), guanidinium thiocyanate (GuSCN), and urea (NH_2_CONH_2_) additives.

**Table 2:** Summary of similar works with different additives and comparison to this work

Author	Fabrication conditions	Fabricating method	Additive treatment	Perovskite	PCE improvement	Operational stability
Muhammad *et al.*^39^	Not reported	One-step	EACl	MAPbI_3_	15%	≈12% loss after 1000 h test in 30% relative humidity in air without encapsulation
Vincent *et al.*^40^	Ambient air	One-step	DIO	MAPbI_3_	60%	Not reported
Nian *et al.*^41^	Nitrogen glovebox	One-step	GuSCN	MAPbI_3_	7%	≈10% loss after 350 h test in ambient humidity in air without encapsulation
Ying *et al.*^42^	Not reported	One-step	NH_2_CONH_2_	MAPbI_3_	11%	Not reported
This work	Ambient air	Sequential deposition	MTP	MAPbI_3_	38%	≈20% loss after 500 h test in 20% relative humidity in dry box without encapsulation

## 4. Conclusion

In this work, we have introduced an efficient method for simultaneous passivation of the grain boundaries and recombination processes in a single cation MAPbI_3_ film using MTP additives to boost the PCE and stability of PSCs. By adding MTP to the MAPbI_3_ harvester layer, we obtained a PCE of 16.93% in mesoporous devices, which is higher than that of untreated devices (12.32%). Without any encapsulation, it was found that MTP-based cells show superior stability performance compared with pristine cells after storage in a humid environment for 500 h. Our findings reveal that 5% of MTP additive can enhance the MAPbI_3_ layer merits, including crystallinity, light absorption, morphology and suppressed residual PbI_2_ while decreasing the recombination rate. Passivating the surfaces and grain boundaries through MTP treatment, the MAPbI_3_ film efficiently prevents the decomposition pathway at the corresponding interfaces. This study will provide insights into the role of MTP additive engineering in the enhancement of PCE and the stability of PSCs.

## Conflicts of interest

The authors declare that they have no conflict of interest.

## Supplementary Material
